# Supplementary material for: Repair of airway epithelia requires metabolic rewiring towards fatty acid oxidation
Source: Nat Commun. 2023 Feb 13;14:721. doi: 10.1038/s41467-023-36352-z (PMC9925445; doi:10.1038/s41467-023-36352-z)
Supplement: Supplementary file 1 — Supplementary Information [file 41467_2023_36352_MOESM1_ESM.pdf]

Supplementary Figure 1

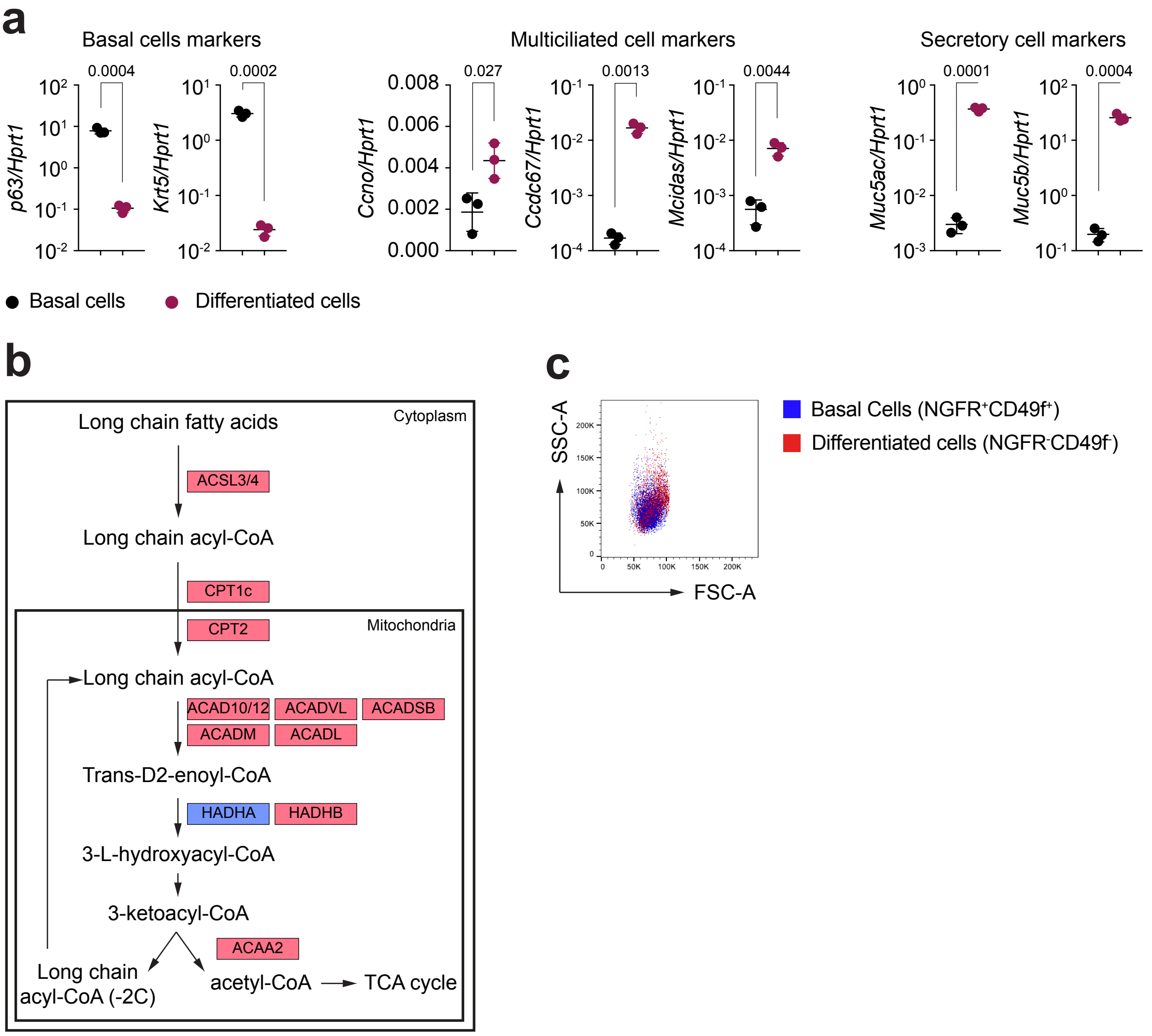

Supplementary Fig. 1. Characterisation of the NGFR$^+$CD49f$^+$ basal and NGFR$^-$CD49f$^-$ progenitors subsets
(a) qPCR analysis of mRNA expression levels of the indicated genes in basal and differentiated cells isolated from mTEC cultures at ALI day 10. Values are normalized to *Hprt1* expression. Data show mean +/- SD. Statistics were performed using two-tailed unpaired Student's t test. *n*=3 independent samples (b) Schematic representation of mitochondrial FAO. In red or blue are highlighted the genes respectively upregulated and downregulated in differentiated cells as compared to basal cells, in the RNA sequencing analysis shown in Fig. 1a-c. (c) Forward scatter area versus side scatter area (FSC vs SSC) gating of sorted CD49f$^+$NGFR$^+$ basal and CD49f$^-$NGFR$^-$ differentiated cells, used for untargeted metabolomics analysis of Fig. 1f-h.

Supplementary Figure 2

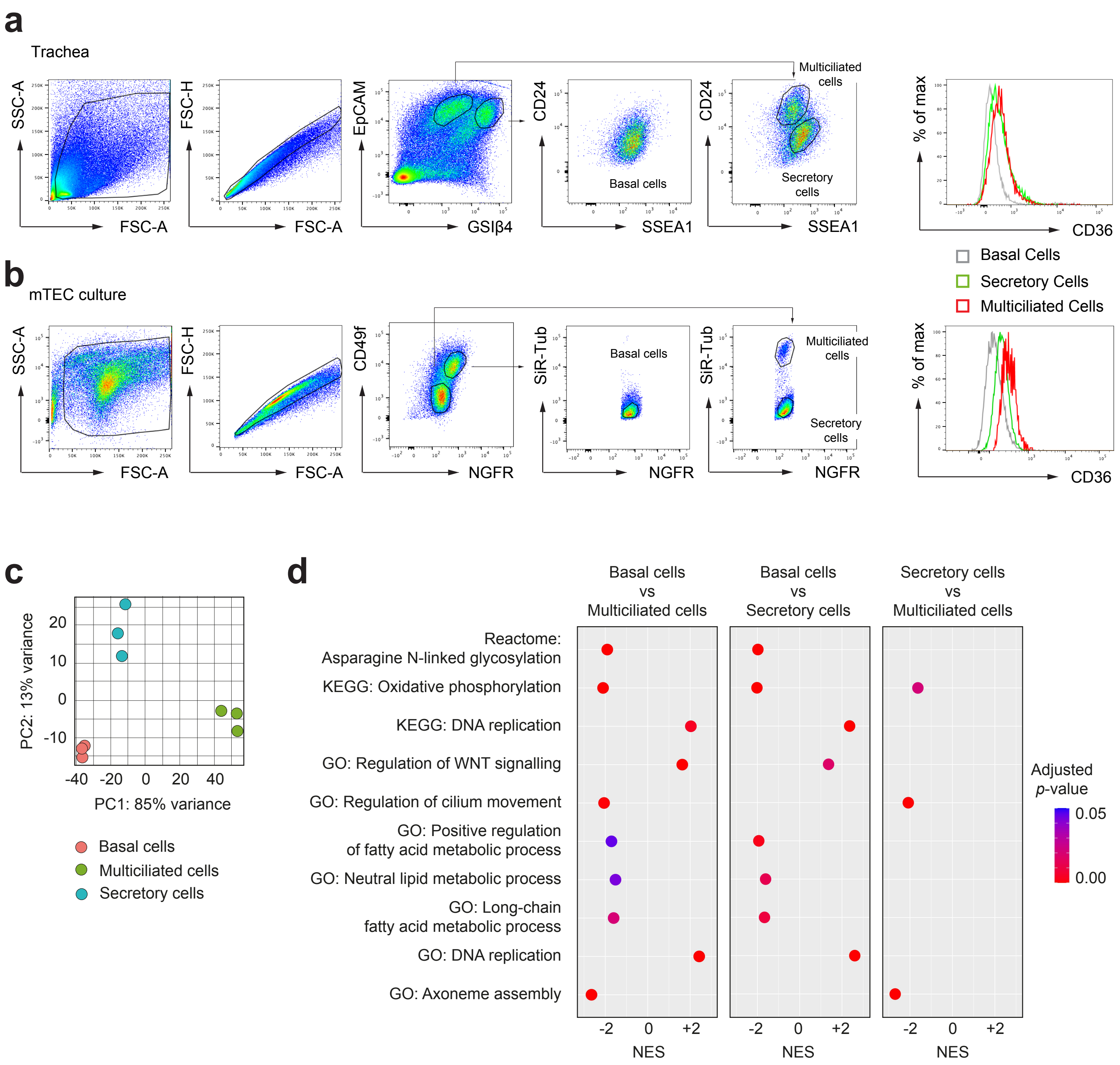

Supplementary Fig. 2. Changes in cellular metabolism accompany differentation of both secretory and multiciliated cells
(**a**) Flow cytometry analysis of ex-vivo airway epithelial cells dissociated from murine tracheae. EpCam+ epithelial cells were separated in GSIβ4^{hi} basal cells, GSIβ4^{low} CD24^{+} SSEA1^{-} ciliated cells, and GSIβ4^{low} CD24^{-} SSEA1^{+} secretory cells[66]. Levels of CD36 expression on the three subpopulations are shown. (**b**) Flow cytometry analysis of mTEC subsets. Before processing for flow cytometry, mTEC cultures were stained with SiR -Tubulin 1 μM for 1 hour at 37°C. Cells were then trypsined and stained with the indicated markers. Levels of CD36 expression on basal (CD49f^{+}NGFR^{+}), secretory (CD49f^{-}NGFR^{-} SiR-tubulin^{-}) and ciliated cells (CD49f^{-}NGFR^{-} SiR-tubulin^{+}) are shown. (**c**) PCA plots of RNA-sequencing data from sorted basal, secretory and ciliated cells, as defined in (b). (**d**) Dot plot representing pathways enriched or reduced in basal, multiciliated and secretory cell, as defined by Gene Set Enrichment Analysis (GSEA). Differential expression analysis was carried out with DESeq2 package (version 1.20.0) within R version 3.5.1. Genes were considered to be differential expressed with p adj < 0.05. Gene Set Enrichment analysis (GSEA, version 2.2.3) was performed using gene lists ranked using the Wald statistics. Gene signatures were considered significant if FDR -value < 0.05. Dot colours relates to statistical significance. NES: normalized enrichment score
.

Supplementary Figure 3

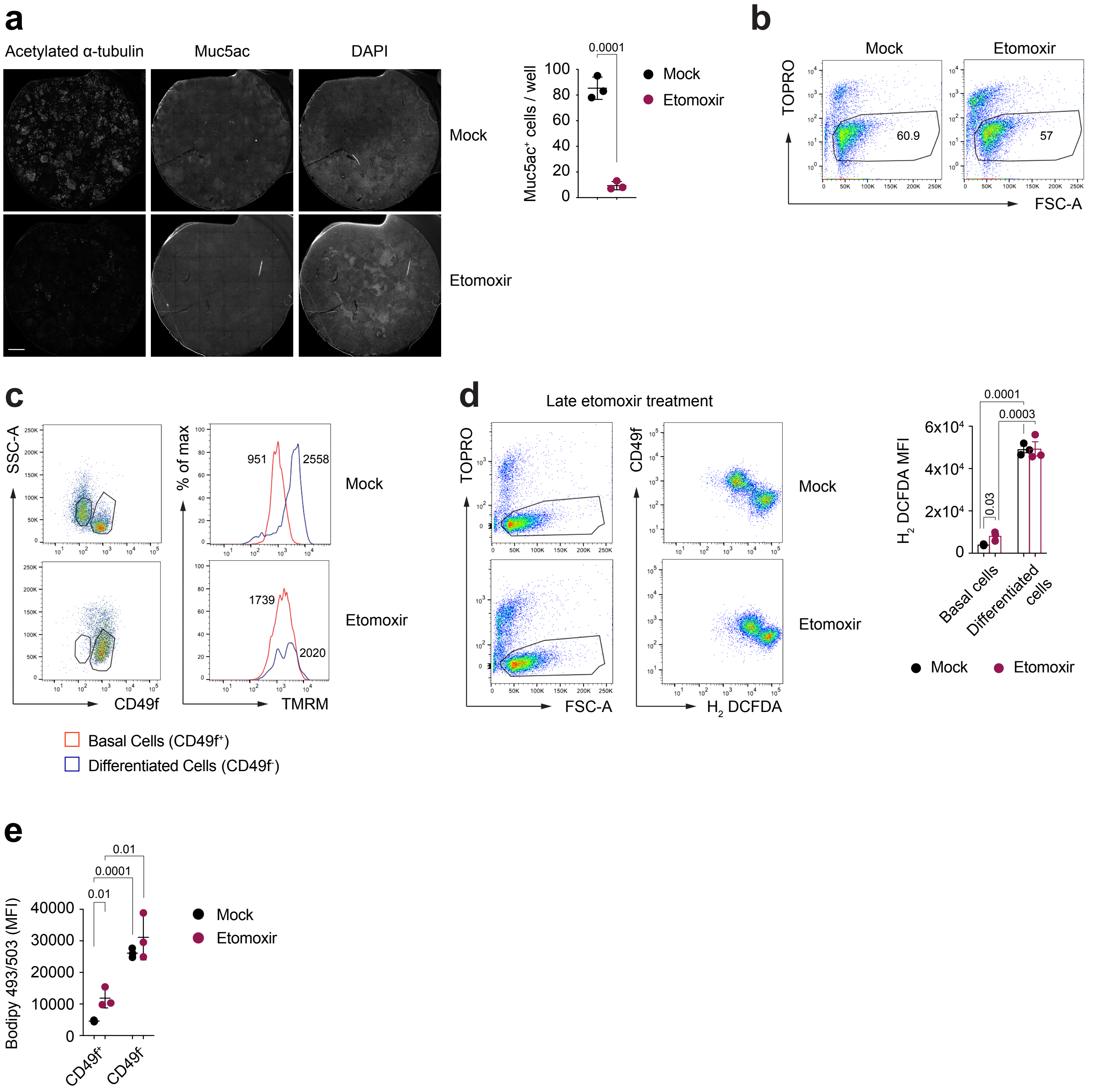

Supplementary Figure 3. Effects of etomoxir on mTEC cultures

(**a**) Immunostaining for acetylated α-tubulin, Muc5ac and DAPI from entire wells of mTEC cultures, grown in presence or absence of etomoxir. Images relate to Figure 2c. Numbers of Muc5ac + cells/wells is also shown. Data show mean +/- SD. Statistics were performed using two-tailed unpaired Student's t test with *n*=3 independent samples (**b**) Flow cytometry analysis of viability of mock or etomoxir-treated mTEC cultures analysed at ALI day 10, as measured by TO-PRO-3 staining. (**c**) Flow cytometry analysis of the mitochondrial transmembrane potential of mock or etomoxir-treated mTEC cultures analysed at ALI day 10, measured by TMRM staining. Numbers next to the histograms indicate TMRM mean fluorescence intensity in basal and differentiated cells. (**d**) Intracellular ROS levels in basal and differentiated from mTEC cultures that were mock treated or treated with etomoxir 50 μM from ALI day 10 till ALI day 12. Data show mean +/- SD. Statistics were performed using two-tailed unpaired Student's t test, *n*=3 independent samples. (**e**) Quantification of intracellular neutral lipids by Bodipy 493/503. MFI: mean fluorescence intensity. Data show mean +/- SD. Statistics were performed using two-tailed unpaired Student's t test. *n*=3 independent samples.

Supplementary Figure 4

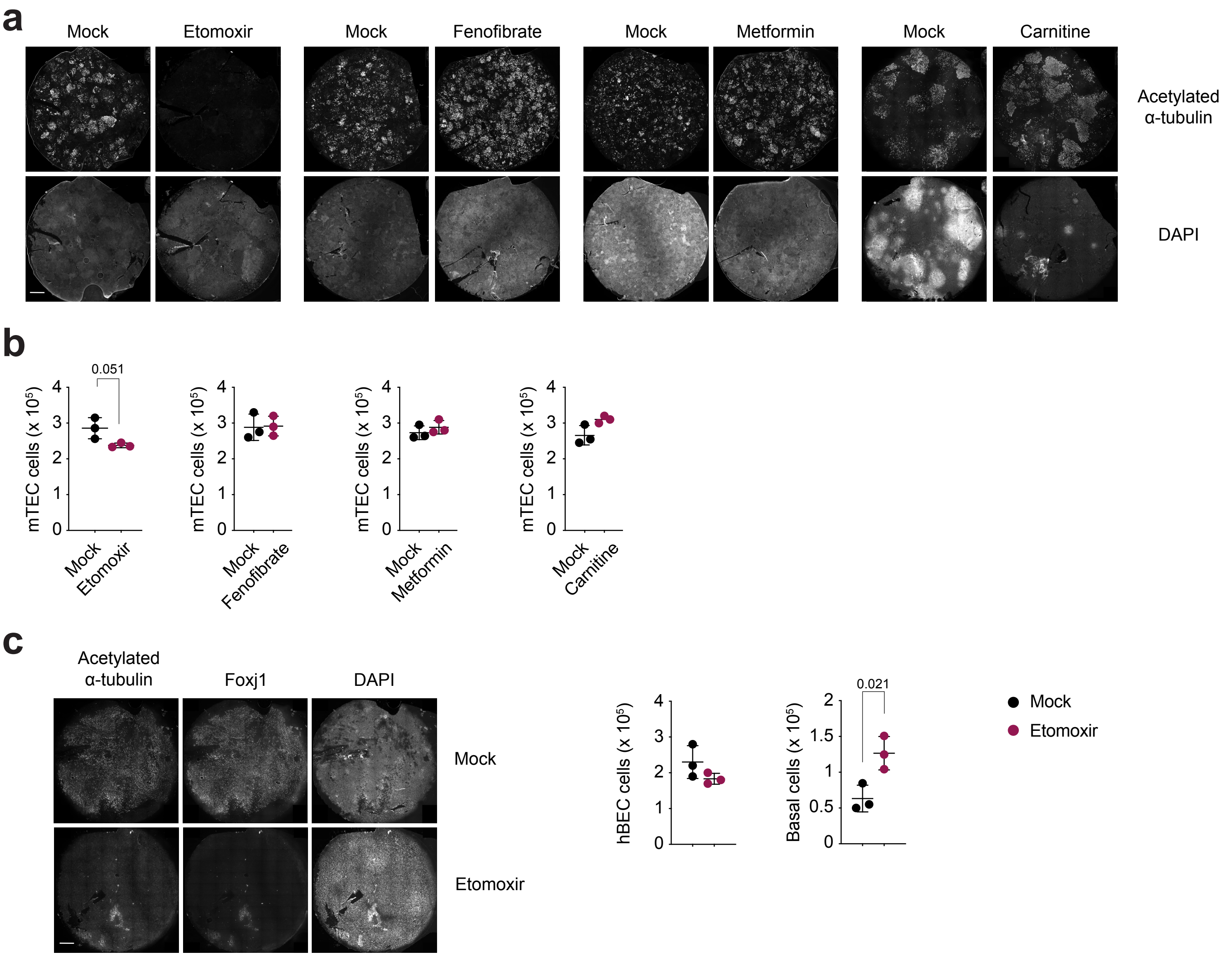

Supplementary Figure 4. Modulation of FAO influences epithelial cell differentation
(**a**) Immunostaining for acetylated α-tubulin and DAPI of mTEC cultures grown in presence or absence of etomoxir, fenofibrate, metformin or carnitine, from the onset of ALI onwards. Cells were analyzed at ALI day 12 and and images relate to the quantifications shown in Fig. 2d. Scale bar: 1 mm (**b**) Cell counts from mTEC cultures treated as indicated in (a) are shown. Data show mean +/- SD. Statistics were performed using two-tailed unpaired Student's t test. *n*=3 independent samples (**c**) Representative immunostaining of acetylated α-tubulin, Foxj1 and DAPI from entire wells of human BEC cultures, grown in presence or absence of etomoxir from ALI day 9 onwards. Images relate to the quantification shown in Fig. 2e. Total cell number and number of $CD49f^+$ $NGFR^+$ basal cells determined by flow cytometry are also shown. Data show mean +/- SD. Statistics were performed using two-tailed unpaired Student's t test. *n*=3 independent samples. Scale bar: 1 mm

Supplementary Figure 5

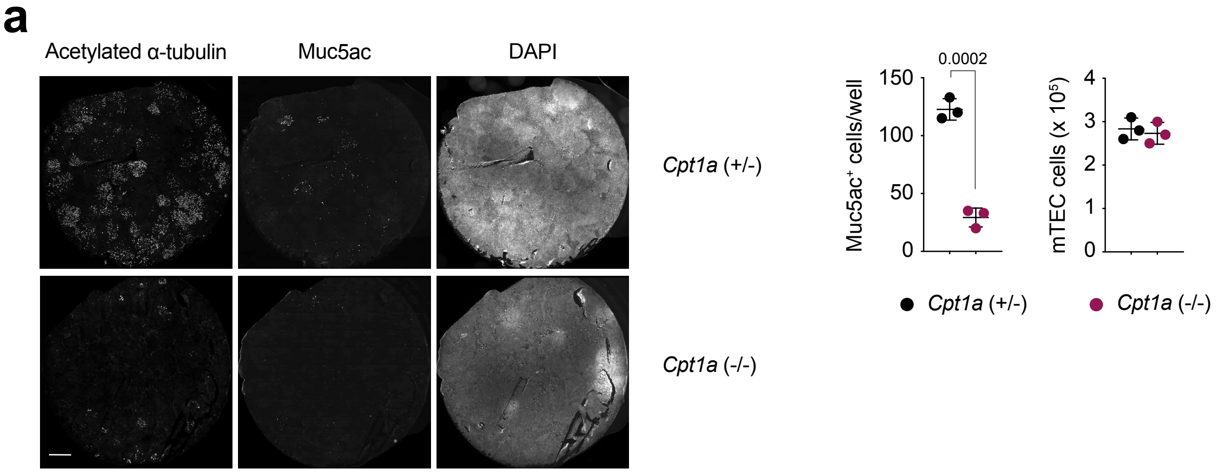

Supplementary Fig. 5. *Cpt1a* deletion prevents airway epithelial differentation.
(**a**) Representative immunostaining for acetylated α-tubulin, Muc5ac and DAPI of *Cpt1a*$^{+/-}$ and *Cpt1a*$^{-/-}$ mTEC cultures at ALI day 10. Number of Muc5ac^{+} cells and total cell count are also shown. Data show mean +/- SD. Statistics were performed using two-tailed unpaired Student's t test. *n*=3 independent samples Quantification of acetylated α-tubulin staining is shown in Figure 3c. Scale bar: 1 mm.

Supplementary Figure 6

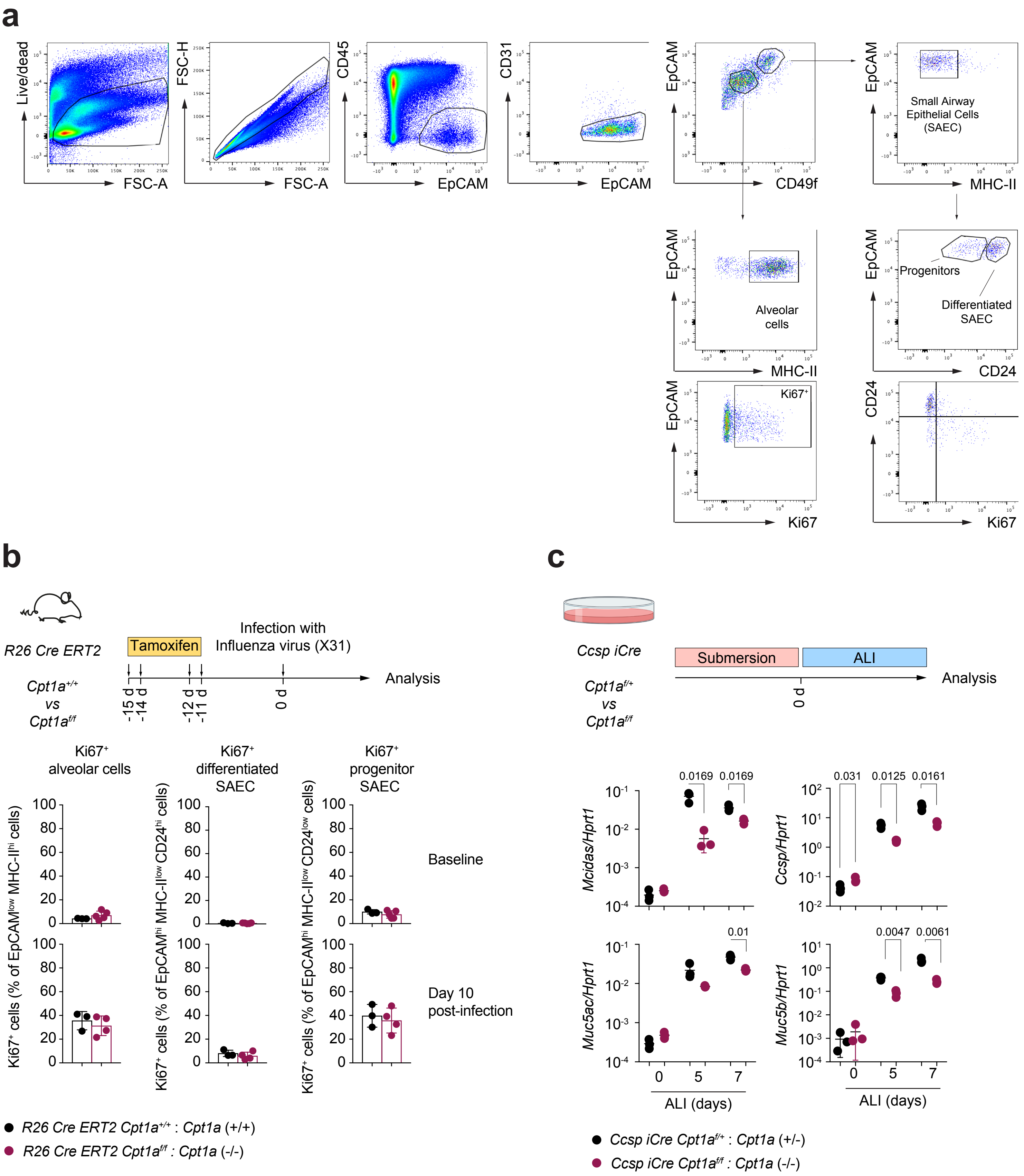

Supplementary Fig. 6. Definition of small airway epithelial cell subsets and analysis of their proliferation rate at baseline and post infection
(**a**) FACS gating strategy to identify alveolar cells, small airway epithelial cells (SAEC), and its specific subsets: progenitor SAEC and differentiated SAEC, in the murine lung. Alveolar cells ($CD45^-$ $CD31^-$ $EpCam^{low}$ $CD49f^{low}$ $MHCII^{hi}$); small airway epithelial cells (SAEC, $CD45^-$ $CD31^-$ $EpCam^{hi}$ $CD49f^{hi}$ $MHCII^{low}$), further subdivided in differentiated small airway epithelia ($CD45^-$ $CD31^-$ $EpCam^{hi}$ $CD49f^{hi}$ $MHCII^{low}$ $CD24^{hi}$) and progenitor cells ($CD45^-$ $CD31^-$ $EpCam^{hi}$ $CD49f^{hi}$ $MHCII^{low}$ $CD24^{low}$). (**b**) Flow cytometry analysis of lungs isolated from *R26 CreERT2 Cpt1a*$^{+/+}$ and *R26 CreERT2 Cpt1a*$^{f/f}$ mice on day 0 or day 10 post-infection to assess the fraction of alveolar cells, progenitor SAEC and differentiated SAEC, as defined in Supplementary Fig. 6a, that are positive for the intranuclear proliferation marker Ki67. Data show mean +/- SD with n = 3-5. Each dot represents an individual mouse. (**c**) qPCR analysis of mRNA expression levels of the indicated genes in mTEC cultures derived from *Ccsp iCre Cpt1a*$^{f/+}$ and *Ccsp iCre Cpt1a*$^{f/f}$ mice at ALI day 0, 5 and 7. Values are normalized to *Hprt1* expression. Data show mean +/- SD. Statistics were performed using multiple unpaired t tests and adjusted using the Holm-Sidak correction method, with n=3 .

Supplementary Figure 7

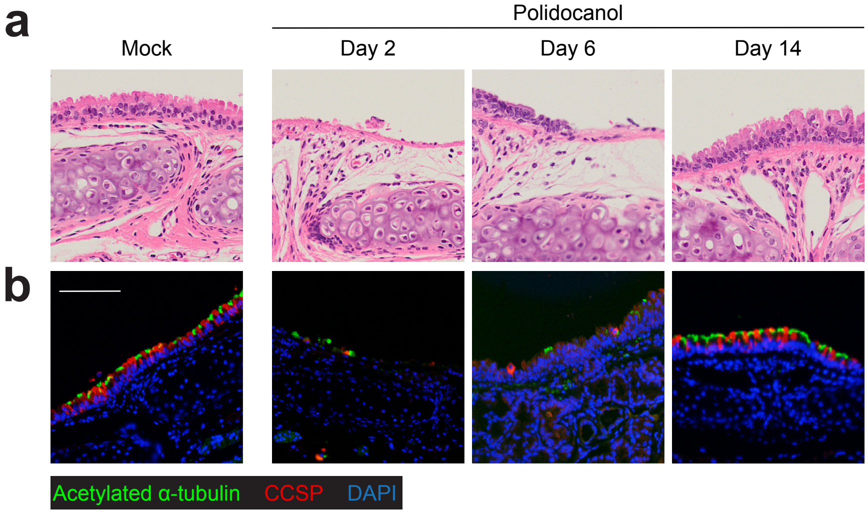

Supplementary Figure 7. Characterisation of the polidocanol-induced tracheal epithelial injury model
(**a**) Haematoxylin & Eosin (H&E) stainings of tracheae from mock or polidocanol-treated *C57Bl/6J* mice at 2, 6, 14 days post injury. (**b**)Sections were stained for acetylated α-tubulin, CCSP and DAPI. Scale bar: 50 μm. Data are representative of *n*=3 independent samples.

Supplementary Figure 8

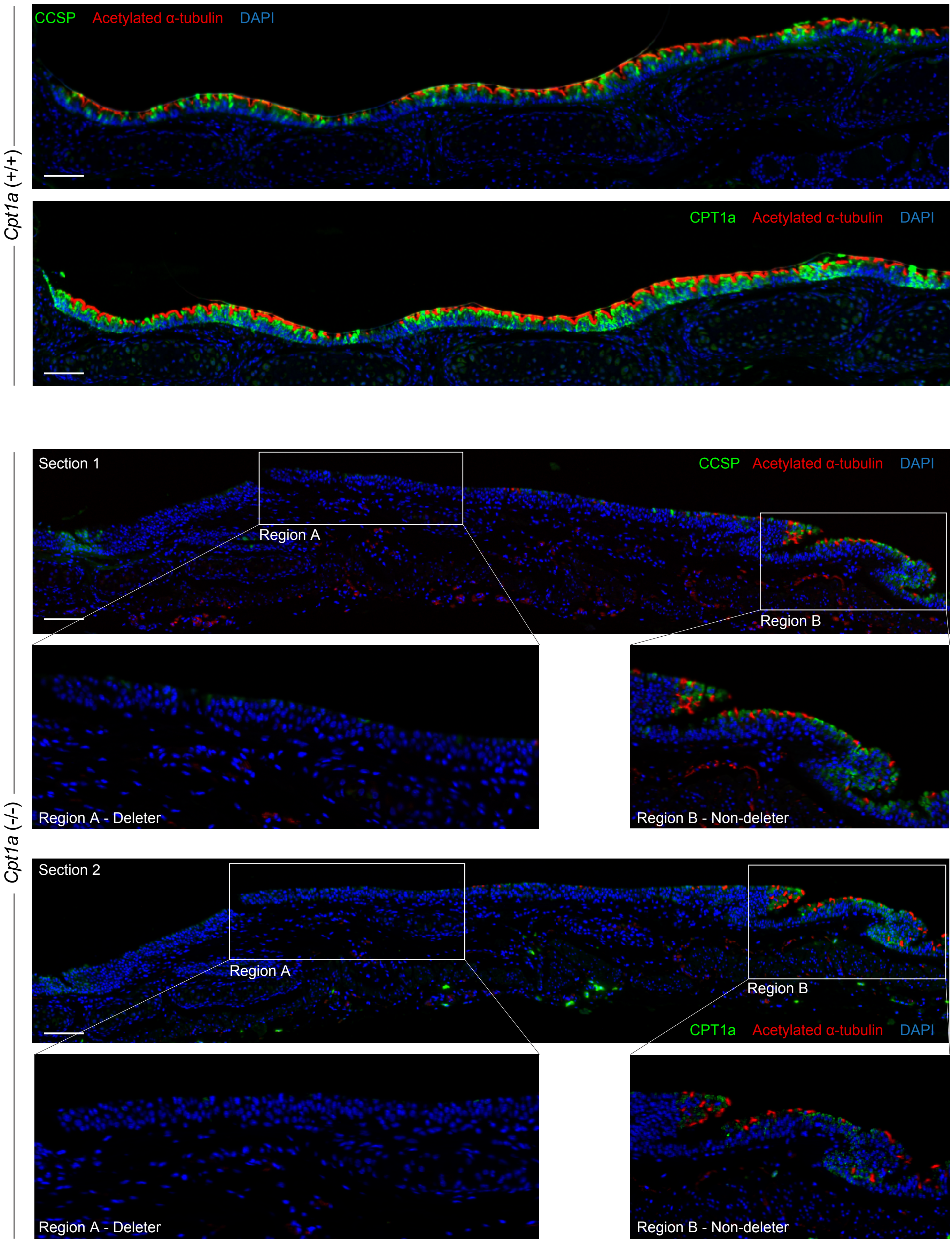

Supplementary Fig. 8. **FAO regulates proximal airway epithelial cell differentiation in vivo**
(a) Fluorescence microscopy analysis of acetylated α-tubulin, CPT1a, CCSP and DAPI from whole tracheae of $Cpt1a^{+/+}$ and $Cpt1a^{-/-}$ mice, 12 days after polidocanol challenge. Boxed images are magnification of the indicated tracheal regions in the low magnification micrograph. Scale bar: 100 μm.
Note that tamoxifen treatment induces only a partial deletion of the Cpt1a gene, so that areas devoid of CPT1a (Region A - Deleter) are adjacent to areas were the CPT1a protein is still detectable (Region B - Non-deleter). Data are representative of three independent experiments with *n*=3 independent samples per experiment.
