# Supplementary material for: Repair of airway epithelia requires metabolic rewiring towards fatty acid oxidation
Source: Nat Commun. 2023 Feb 13;14:721. doi: 10.1038/s41467-023-36352-z (PMC9925445; doi:10.1038/s41467-023-36352-z)
Supplement: Supplementary file 3 — Reporting Summary [file 41467_2023_36352_MOESM3_ESM.pdf]

nature portfolio

Corresponding author(s): Andreas Wack

Last updated by author(s): 23/1/23

Reporting Summary

Nature Portfolio wishes to improve the reproducibility of the work that we publish. This form provides structure for consistency and transparency in reporting. For further information on Nature Portfolio policies, see our Editorial Policies and the Editorial Policy Checklist.

Statistics

For all statistical analyses, confirm that the following items are present in the figure legend, table legend, main text, or Methods section.

n/a	Confirmed	
☐	☒	The exact sample size (*n*) for each experimental group/condition, given as a discrete number and unit of measurement
☐	☒	A statement on whether measurements were taken from distinct samples or whether the same sample was measured repeatedly
☐	☒	The statistical test(s) used AND whether they are one- or two-sided<br>*Only common tests should be described solely by name; describe more complex techniques in the Methods section.*
☒	☐	A description of all covariates tested
☐	☒	A description of any assumptions or corrections, such as tests of normality and adjustment for multiple comparisons
☐	☒	A full description of the statistical parameters including central tendency (e.g. means) or other basic estimates (e.g. regression coefficient) AND variation (e.g. standard deviation) or associated estimates of uncertainty (e.g. confidence intervals)
☐	☒	For null hypothesis testing, the test statistic (e.g. *F*, *t*, *r*) with confidence intervals, effect sizes, degrees of freedom and *P* value noted<br>*Give P values as exact values whenever suitable.*
☒	☐	For Bayesian analysis, information on the choice of priors and Markov chain Monte Carlo settings
☐	☒	For hierarchical and complex designs, identification of the appropriate level for tests and full reporting of outcomes
☒	☐	Estimates of effect sizes (e.g. Cohen's *d*, Pearson's *r*), indicating how they were calculated

Our web collection on statistics for biologists contains articles on many of the points above.

Software and code

Policy information about availability of computer code

Data collection	N/A
Data analysis	RNA-seq (references included in materials and methods):<br>Read quality trimming and adaptor removal was carried out using Trimmomatic (version 0.36)<br>Reads were aligned to the mouse genome (Ensembl GRCm38 release 89) using STAR (version 2.5.2a)(51) and gene level counts were obtained using the RSEM package (version 1.3.0)<br>Differential expression analysis was carried out with DESeq2 package (version 1.24.0)53 within R version 3.6.0(54)<br>Gene Set Enrichment analysis (GSEA, version 2.2.3)(55) was performed for each pairwise comparison using gene lists ranked using the Wald statistic<br>Over enrichment analyses was performed using Qiagen's IPA software<br>To investigate differences in lipid composition, we quantile normalized the complete lipidomics data set using R function "normalize.quantiles ()" from package preprocessCore (Bolstad, B.M. preprocessCore: A collection of pre-processing functions.<br>R Package Version 1.50.0, 2020 doi:10.18129/B9.bioc.preprocessCore).

For manuscripts utilizing custom algorithms or software that are central to the research but not yet described in published literature, software must be made available to editors and reviewers. We strongly encourage code deposition in a community repository (e.g. GitHub). See the Nature Portfolio guidelines for submitting code & software for further information.

Data

Policy information about availability of data

All manuscripts must include a data availability statement. This statement should provide the following information, where applicable:

- Accession codes, unique identifiers, or web links for publicly available datasets
- A description of any restrictions on data availability
- For clinical datasets or third party data, please ensure that the statement adheres to our policy

Sequencing data are available in GEO under accession code GSE209686 and GSE218663 .

Human research participants

Policy information about studies involving human research participants and Sex and Gender in Research.

Reporting on sex and gender	N/A
Population characteristics	N/A
Recruitment	N/A
Ethics oversight	N/A

Note that full information on the approval of the study protocol must also be provided in the manuscript.

Field-specific reporting

Please select the one below that is the best fit for your research. If you are not sure, read the appropriate sections before making your selection.

[x] Life sciences [ ] Behavioural & social sciences [ ] Ecological, evolutionary & environmental sciences

For a reference copy of the document with all sections, see nature.com/documents/nr-reporting-summary-flat.pdf

Life sciences study design

All studies must disclose on these points even when the disclosure is negative.

Sample size	No statistical tests were used to predetermine sample size. At least three biological replicates were included per independent experiment.
Data exclusions	For in vivo influenza virus infection studies, mice were excluded if the intranasal infection was not efficient (i.e., mice partially expelled the diluted virus while anaesthetised), did not display any clinical scores and if they did not lose weight (<3% of total body weight loss) on day 6 post infection.
Replication	All experiments were replicated at a minimum of two times, but mostly 3-4 times.<br>All single data points in all graphs in all figures represent biological replicates, i.e. samples derived from separate mice, or, in the case of primary murine and human epithelial cells, measurements were performed on independently grown cultures.
Randomization	Mice of similar age and identical sex were randomly assigned to groups prior to the initiation of the experiment.
Blinding	The investigators were not blinded to experimental conditions, as all experiments were conducted and analysed by one person. Sequencing analysis was performed by a bioinformatician who had no relevant prior knowledge of the biological conditions, and therefore performed analysis in an unbiased fashion.

Reporting for specific materials, systems and methods

We require information from authors about some types of materials, experimental systems and methods used in many studies. Here, indicate whether each material, system or method listed is relevant to your study. If you are not sure if a list item applies to your research, read the appropriate section before selecting a response.

Materials & experimental systems

n/a	Involved in the study
☐	☒ Antibodies
☐	☒ Eukaryotic cell lines
☒	☐ Palaeontology and archaeology
☐	☒ Animals and other organisms
☒	☐ Clinical data
☒	☐ Dual use research of concern

Methods

n/a	Involved in the study
☒	☐ ChIP-seq
☐	☒ Flow cytometry
☒	☐ MRI-based neuroimaging

Antibodies

Antibodies used

EpCam-APC, Clone: G8.8. Invitrogen. CAT: 17-5791-82. Concentration: 2.5 µg/ml.
CD45-BV786, Clone: 30-F11. BioLegend. CAT: 103149. Concentration: 2.5 µg/ml.
CD31-BV421, Clone: RM5228. Invitrogen. CAT: 103149. Concentration: 2.5 µg/ml.
CD24-BV510, Clone: M1/69. BioLegend. CAT: 101831. Concentration: 10 µg/ml.
MHC-II-BV711, Clone: M5/114.15.2. BioLegend. CAT: 107643. Concentration: 2 µg/ml.
GSIb4-bio, isolectin, Invitrogen, CAT: I21414 Concentration 5 µg/ml.
SSEA1-PE, Clone: eBio MC-480. eBioscience. CAT: 12-8813-42 Dilution:1:50
Fixable blue dead stain-BUV395. ThermoFisher. CAT: L23105. Concentration: 1 µl/10^6 cells.
CD36-APC, Clone HM36, BioLegend, CAT:102612. Concentration: 2 µg/ml.
CD49f-PECy7, Clone: GeH3, eBioscience, CAT: 25-0495-82 Concentration: 1 µg/ml.
NGFR-AF488, rabbit polyclonal, Atsbio , CAT: FL-N01AP, Concentration: 1 µg/ml
Ki67-PE, Clone SolA15, eBioscience, CAT: 12-5698-80 Concentration: 1 µg/ml
CPT1a, rabbit polyclonal, Proteintech, CAT: 15184-1-AP, Concentration: 1 µg/ml
CCSP, rabbit polyclonal, Abcam, CAT: ab40873, Dilution: 1:500
acetylated-alpha tubulin, Clone:6-11B-1, Sigma, CAT: T7451, Dilution: 1:1000
Foxj1, clone 2A5, eBioscience, CAT:14-9965, Dilution 1:500

Goat anti-rabbit AF488, polyclonal (H+L). ThermoFisher. CAT: A11034. 1/400 dilution.
Goat anti-rabbit AF568, polyclonal (H+L). ThermoFisher. CAT: A11011. 1/400 dilution.
Goat anti-mouse IgG2b AF488, polyclonal (H+L). ThermoFisher. CAT: A21141. 1/400 dilution.
Goat anti-mouse IgG2b AF568, polyclonal (H+L). ThermoFisher. CAT: A21144. 1/400 dilution.
Goat anti-rabbit- HRP, polyclonal (H+L). Biorad. CAT:170-6515. 1/5000 dilution
Beta-actin, rabbit monoclonal , Clone: 13E5, Cell Signaling Tech, CAT: 4970. 1/5000 dilution

Validation

Antibodies were all sourced commercially with independent validations and citations.

Eukaryotic cell lines

Policy information about cell lines and Sex and Gender in Research

Cell line source(s)

Primary human NHBE- bronchial epithelial cells were purchased from Lonza cat n. CC-2540

Authentication

No authentication procedure performed

Mycoplasma contamination

All cell tested negative for mycoplama

Commonly misidentified lines
(See ICLAC register)

n/a

Animals and other research organisms

Policy information about studies involving animals; ARRIVE guidelines recommended for reporting animal research, and Sex and Gender in Research

Laboratory animals

Mice were aged 8-12 weeks at the start of experiments.

Strains (references in manuscript):
All genotypes were bred on a C57BL/6J background.
Cpt1a fl/fl (Cpt1atm1.1Pec) were crossed to mice carrying a tamoxifen-inducible CreERT2 in the ROSA26 locus (Gt(ROSA)26Sortm1 (cre/ERT2)Thl);
or mice carrying iCre recombinase in the Ccsp/Scgb1a1 locus (Scgb1a1tm1(icre)Fjd).

Wild animals	N/A
Reporting on sex	Both male and female mice were used throughout the study - using sex-matched groups for each experiment.
Field-collected samples	N/A
Ethics oversight	Francis Crick Institute in accordance with approval by the Home Office, UK (project license: P9C468066)

Note that full information on the approval of the study protocol must also be provided in the manuscript.

Flow Cytometry

Plots

Confirm that:

- [ ] The axis labels state the marker and fluorochrome used (e.g. CD4-FITC).
- [x] The axis scales are clearly visible. Include numbers along axes only for bottom left plot of group (a 'group' is an analysis of identical markers).
- [x] All plots are contour plots with outliers or pseudocolor plots.
- [x] A numerical value for number of cells or percentage (with statistics) is provided.

Methodology

Sample preparation	For cell isolation from lung tissues, mice were euthanised (600mg kg-1 pentobarbital/17mg kg-1 mepivacaine) and then perfused with 10 ml of ice-cold PBS through the right ventricle of the heart. 1.5 ml Dispase II (Roche) (5 mg/ml in IMDM) was then injected intratracheally into the lungs, followed by 0.4 ml 1% low-gelling agarose solution (in PBS) (Sigma). Mice were then placed on ice allowing the agarose/dispase-filled lungs to set. Lungs were then dissected and placed in 2 ml Dispase II solution for 30 minutes to dissociate epithelial cells. Lungs were passed through a 100 um filter, before a 10-minute DNase I digestion (50µg/ml) (Sigma). Following digestion, lung homogenates were passed through a 70 um filter and centrifuged at 1,400r.p.m. for 5min at 4°C, before red blood cell lysis. Single cell suspensions were preincubated with anti-FcgRIII/II (Fc block), before a 30-min incubation with the indicated fluorochrome-labelled antibodies. For Ki67 staining, cells were fixed and permeabilised before staining.<br>Airway epithelial cells from trachea were dissociated using Dispase II (Roche) (5 mg/ml in IMDM), for 2 hours at 37°C. After incubation, dissociated tissues were washed in media containing 10% FCS, passed through a 70 um strainer, pelleted and stained with the indicated antibody panel for flow cytometry.
Instrument	Cells were analysed on a BD LSRFortessa cell analyser (BD Bioscience).
Software	Flow data was interpreted using the software FlowJo v10.6.2 (FlowJo, RRID:SCR_008520).
Cell population abundance	FACS-isolated populations had a purity of >98% as determined by FACS purity check.
Gating strategy	Gating strategy is in Fig. 1a and Supplementary Fig. 2b for RNA seq of sorted basal and differentiated (secretory and ciliated) cells. Supplementary Fig. 6a shows gating strategy for lung-derived cell populations.

- [x] Tick this box to confirm that a figure exemplifying the gating strategy is provided in the Supplementary Information.
